# Gene expression profiling of tumours derived from ras^V12^/E1A-transformed mouse embryonic fibroblasts to identify genes required for tumour development

**DOI:** 10.1186/1476-4598-4-4

**Published:** 2005-01-16

**Authors:** Sophie Vasseur, Cédric Malicet, Ezequiel L Calvo, Jean Charles Dagorn, Juan L Iovanna

**Affiliations:** 1 INSERM U.624, Stress Cellulaire, 163 Avenue de Luminy, Case 915, Parc Scientifique et Technologique de Luminy, 13288 Marseille Cedex 9, France

**Keywords:** ras, E1A, MEF, microarray, gene expression, tumour development.

## Abstract

**Background:**

In cancer, cellular transformation is followed by tumour development. Knowledge on the mechanisms of transformation, involving activation of proto-oncogenes and inactivation of tumour-suppressor genes has considerably improved whereas tumour development remains poorly understood. An interesting way of gaining information on tumour progression mechanisms would be to identify genes whose expression is altered during tumour formation. We used the Affymetrix-based DNA microarray technology to analyze gene expression profiles of tumours derived from ras^V12^/E1A-transformed mouse embryo fibroblasts in order to identify the genes that could be involved in tumour development.

**Results:**

Among the 12,000 genes analyzed in this study, only 489 showed altered expression during tumour development, 213 being up-regulated and 276 down-regulated. The genes differentially expressed are involved in a variety of cellular functions, including control of transcription, regulation of mRNA maturation and processing, regulation of protein translation, activation of interferon-induced genes, intracellular signalling, apoptosis, cell growth, angiogenesis, cytoskeleton, cell-to-cell interaction, extracellular matrix formation, metabolism and production of secretory factors.

**Conclusions:**

Some of the genes identified in this work, whose expression is altered upon ras^V12^/E1A transformation of MEFs, could be new cancer therapeutic targets.

## Background

Cellular transformation is a complex process which involves activation of proto-oncogenes and inactivation of tumour-suppressor genes [1]. After transformation, the cells can generate malignant tumours, by mechanisms only partly understood yet. It is supposed that some modifications in the pattern of gene expression will promote survival of transformed cells *in situ*, other modifications will favour eventual formation of metastases [2], the capacity to adapt a new microenvironment being of major importance for successful tumour development and progression [reviewed in [3]]. Therefore, identification of genes whose expression is altered during tumour formation should provide important information on the underlying molecular mechanisms. In the present work, we used the Affymetrix-based DNA microarray technology to analyze gene expression profiles of tumour-derived from ras^V12^/E1A-transformed primary mouse embryonic fibroblasts (MEFs), in order to identify genes associated with tumour development.

## Results and Discussion

**Figure 1:**
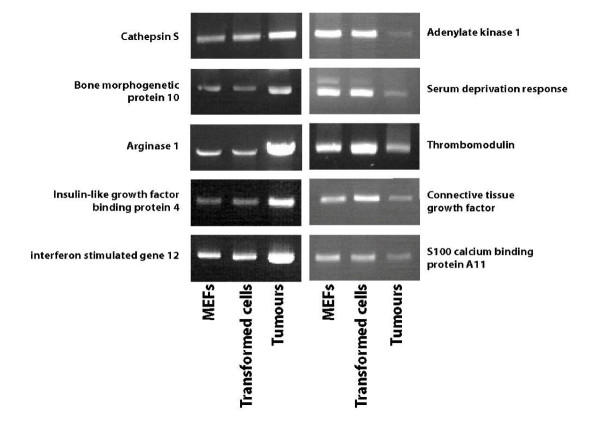
Confirmation of microarray results by sequantitative RT-PCR analysis. Total RNA was isolated from primary embryo fibroblasts (MEFs), ras^V12^/E1A MEFs and ras^V12^/E1A MEF-induced tumours. In these three preparations, mRNA encoding arginase 1, bone morphogenetic protein 10, cathepsin S, insulin-like growth factor binding protein 4, interferon stimulated gene 12, serum deprivation response, thrombomodulin, adenylate kinase 1, connective tissue growth factor and S100 calcium binding protein A11 were amplified by RT-PCR as described in Material and Methods section.

The *ras *oncogene can transform most immortalized rodent cells to generate tumour cells, whereas transformation of primary cells requires either a cooperating oncogene or the inactivation of a tumour suppressor gene. The adenovirus E1A oncogene cooperates with *ras *to transform primary rodent fibroblasts [4] and injection of athymic mice with such transformed fibroblasts induces tumour development. The ras^V12^/E1A model of tumour formation was used in this work to analyze genes necessary for tumour progression. This model was chosen because transformation is induced in a simple and controlled way, avoiding the difficulties of analyzing the multiple and complex transformation mechanisms observed in cellular models derived from human tumours. Mouse embryo fibroblasts (MEFs) were chosen for transformation by ras^V12^/E1A to keep the model homogeneous, the host being the athymic mouse. Because non-transformed MEFs are unable to induce tumour when injected into athymic mice, we previously analyzed the change in gene expression profile induced in MEFs by ras^V12^/E1A-transformation [5], the idea being that such genetic changes are, directly or indirectly, responsible for the capacity of transformed MEFs to form tumours upon injection. As a follow-up, we used in this work microarray analysis to compare expression profiles of about 12,000 genes in ras^V12^/E1A-transformed MEFs and in the tumours formed after their injection into nu/nu mice. With Affymetrix microarray technology, differential expression values greater than 1.7 are likely to be significant, based on internal quality control data. We present data that use a more stringent ratio, restricting our analysis to genes overexpressed or under-expressed at least 2.0 fold in tumours, compared to the parent ras^V12^/E1A-transformed fibroblasts. Most striking findings are summarized below while complete data are presented in Tables 1 and 2 (see additional files 1 and 2), values being the average of three separate experiments. Among the 12,000 genes analyzed in this study, only 489 (4%) showed altered expression upon tumour development. Whereas 213 were up-regulated, 276 were down-regulated. Sixty seven genes encode ESTs. For 10 genes, expression data from microarrays were confirmed (Figure 1) by semiquantitative RT-PCR (see Material and Methods).

It is noteworthy that, to form tumours, transformed cells require the vicinity of blood vessels and components of the stroma, fibroblasts and inflammatory cells. Consequently, mRNA quantified in our system will come from transformed cells growing within the tumour and from stromal cells provided by the host. For example, mRNAs for haemoglobin, selectin or immunoglobulin heavy chain (V10 family) will very probably originate from erythrocytes, endothelium and leucocytes respectively.

### Gene transcription, mRNA processing and translation-associated genes

Down-regulation was observed for some DNA-binding proteins and transcriptional factors such as zinc finger protein 36, C3H type-like 2, forkhead box M1, T-box 14, SET and MYND domain containing 2, sine oculis-related homeobox 1 homolog (Drosophila), liver-specific bHLH-Zip transcription factor, deformed epidermal autoregulatory factor 1 homolog (Drosophila), HMG box Bromodomain (5 domains) Zinc finger C2H2 type, BTB (POZ) domain containing 14A, Jun oncogene, general transcription factor III A, MYB binding protein (P160) 1a, general transcription factor II H polypeptide 1, transcription factor Dp 1, myeloblastosis oncogene-like 2, general transcription factor IIF polypeptide 1, v-ets erythroblastosis virus E26 oncogene homolog 1 (avian), transcriptional regulator SIN3B homolog (yeast), RNA polymerase I transcription factor RRN3, Kruppel-like factor 5, general transcription factor III C 1, nucleosome assembly protein 1-like 1, general transcription factor IIB and transformation related protein 53 (p53) whereas CCR4-NOT transcription complex subunit 7, transcription factor 15, dimerization cofactor of hepatocyte nuclear factor 1 alpha (TCF1), chromobox homolog 3 (Drosophila HP1 gamma), ets variant gene 1, basic helix-loop-helix domain containing class B2, pre-B-cell colony-enhancing factor 1, homeo box C6, BTB (POZ) domain containing 1, zinc finger protein 37, enhancer of zeste homolog 1 (Drosophila) and AT rich interactive domain 3B (Bright like) were up-regulated.

A number of genes involved in RNA maturation, protein translation, processing and secretion were down-regulated in tumours, such as eukaryotic translation initiation factor 4 gamma 1, ER degradation enhancer mannosidase alpha-like 1, paraspeckle protein 1, CUG triplet repeat, RNA binding protein 1, copper chaperone for superoxide dismutase, splicing factor arginine/serine-rich 2 interacting protein, ribosomal protein S6 kinase polypeptide 4, sorting nexin 14, sorting nexin 17, elongation protein 3 homolog (S. cerevisiae), eukaryotic translation initiation factor 5B, peptidylprolyl isomerase F (cyclophilin F), ubiquitin-conjugating enzyme E2S, DnaJ homolog subfamily C member 3, SEC14-like 1 (S. cerevisiae), mitochondrial ribosomal protein L18, mitochondrial isoleucine tRNA synthetase, brix domain-containing protein, proteasome 26S subunit non-ATPase 1, COP9 subunit 5, small nuclear ribonucleoprotein polypeptide A, heat shock protein 105, heat shock protein 1, ribosome-binding protein p34, splicing factor 3b subunit 1, proteasome 26S subunit non-ATPase 7, mitochondrial processing peptidase beta, translocating chain-associating membrane protein 1, mitochondrial ribosomal protein L44, mitochondrial ribosomal protein S25, mitochondrial ribosomal protein S10, eukaryotic translation initiation factor 3 subunit 1 alpha, eukaryotic translation initiation factor 1A, COP9 subunit 2, RNA-binding region (RNP1, RRM) containing 1, DNAJ domain-containing and Der1-like family protein, whereas only 7 genes from this group were up-regulated including ubiquitin specific protease 18, proteosome subunit beta type 8, heterogeneous nuclear ribonucleoprotein U, ERO1-like (S. cerevisiae), proteasome subunit beta type 10, hnRNP-associated with lethal yellow and peptidylprolyl isomerase (cyclophilin)-like 2.

Altogether these results show that transcriptional factors and DNA-binding proteins involved in transcription, as well as proteins involved in RNA maturation, protein translation, processing and secretion are preferentially down regulated during tumour development, suggesting that protein synthesis is less active in tumours than in transformed cells *in vitro*.

### Interferon-induced genes

It is interesting to note that 11 genes activated by interferon were found up-regulated in tumours from 3.2 to 44.6 folds. They comprise the interferon stimulated gene 12 and genes encoding the interferon-induced protein with tetratricopeptide repeats 1, the interferon-induced protein with tetratricopeptide repeats 3, the interferon regulatory factor 7, the interferon alpha-inducible protein, the interferon consensus sequence binding protein 1, the interferon-inducible GTPase, the interferon induced transmembrane protein 2, the interferon gamma induced GTPase, the interferon-g induced GTPase and the interferon gamma-inducible protein 16

### Genes encoding signalling factors

Expression of several genes involved in signalling was modified in tumours. Among up-regulated were genes encoding the TYRO protein tyrosine kinase binding protein, ADP-ribosylation factor-like 4, ARF-GAP RHO-GAP ankyrin repeat and pleckstrin homology domains-containing protein 3, guanosine diphosphate dissociation inhibitor 2, SET domain bifurcated 1, acid phosphatase 5, guanylate nucleotide binding protein 3, SH3-domain GRB2-like 2, rap2 interacting protein x, TGFB inducible early growth response 1, pleckstrin homology domain containing family A (phosphoinositide binding specific) member 1, A kinase (PRKA) anchor protein 8, PDZ and LIM domain 4, protein tyrosine phosphatase non-receptor type 13, calcium and integrin binding family member 2, diaphanous homolog 2 (Drosophila), SH3-domain GRB2-like 3, Janus kinase 3, dual specificity phosphatase 9, Nik related kinase, synaptojanin 2, zinc finger RAN-binding domain containing 1, inositol hexaphosphate kinase 1, spleen tyrosine kinase, ribitol kinase putative, B lymphoid kinase, DEAD box polypeptide 27, mitogen activated protein kinase kinase kinase kinase 1, tumor-associated calcium signal transducer 1 and ral guanine nucleotide dissociation stimulator. Among down-regulated during tumour development where genes encoding adenylate kinase 1, serum deprivation response, ATP-binding cassette sub-family F member 2, protein kinase C alpha, RIO kinase 1 homolog (yeast), protein phosphatase 1 regulatory (inhibitor) subunit 2, phosphotidylinositol transfer protein beta, RAN GTPase activating protein 1, v-crk sarcoma virus CT10 oncogene homolog (avian)-like, serum/glucocorticoid regulated kinase, DEAD box polypeptide 48, nucleolar GTPase, calcium/calmodulin-dependent protein kinase II delta, cellular retinoic acid binding protein I, AFG3(ATPase family gene 3)-like 1 homolog (yeast), transforming growth factor beta regulated gene 4, enabled homolog (Drosophila), Rho GDP dissociation inhibitor gamma, S100 calcium binding protein A11 (calizzarin), cornichon homolog (Drosophila), butyrate-induced transcript 1, phosphatidylinositol 3-kinase regulatory subunit polypeptide 1 (p85 alpha), mitogen activated protein kinase 1, IQ motif containing GTPase activating protein 1, protein kinase C epsilon, transducin (beta)-like 3, serine/threonine kinase 16, thymoma viral proto-oncogene 1, ATP-binding cassette sub-family B (MDR/TAP) member 7, protein phosphatase 1 regulatory (inhibitor) subunit 7, dual specificity phosphatase 1, Rho-associated coiled-coil forming kinase 2, acid phosphatase 1 soluble, PAK1 interacting protein 1, RAN binding protein 1, dual specificity phosphatase 16, RAB23 member RAS oncogene family, WD repeat domain 26, PDZ and LIM domain 1 (elfin) and mitogen activated protein kinase kinase 4.

### Apoptosis-related genes

The ras^V12^/E1A-transformed MEFs are very sensitive to apoptosis *in vitro *[6] whereas, on the contrary, these cells do not show signs of apoptosis in tumours as judged by microscopic analysis. A possible explanation is provided by the observation that many proapoptotic genes are down-regulated in tumours, such as growth arrest and DNA-damage-inducible 45 beta, wild-type p53-induced gene 1, Bcl2-associated X protein, programmed cell death 2, TP53 apoptosis effector, programmed cell death 6 interacting protein, apoptotic chromatin condensation inducer 1, cell division cycle and apoptosis regulator 1, large tumour suppressor 2 and caspase 7 and several antiapoptotic genes are up-regulated, including genes encoding the spermatogenesis apoptosis-related protein and BCL2/adenovirus E1B 19kDa-interacting protein 1 (NIP3).

### Cell growth-involved genes

Another interesting point to be underscored is that expression of many cell growth-related genes was found decreased in tumours, whereas none of them was up-regulated. Among up-regulated genes were those coding for he bone morphogenetic protein 10, cytokine receptor-like factor 1, insulin-like growth factor binding protein 4, ephrin A2, schlafen 4, early growth response 2, retinoblastoma-associated factor 600, receptor tyrosine kinase-like orphan receptor 2, cyclin-dependent kinase 7 (homolog of Xenopus MO15 cdk-activating kinase), inhibitor of growth family member 4 and neoplastic progression 1 were up-regulated and connective tissue growth factor, ephrin B2, neural proliferation differentiation and control gene 1, nerve growth factor beta, Eph receptor A2, neoplastic progression 3, cyclin G1, bone morphogenetic protein receptor type 1A, cell division cycle 34 homolog (S. cerevisiae), cyclin-dependent kinase inhibitor 1A (p21), SGT1 suppressor of G2 allele of SKP1 homolog (S. cerevisiae), nuclear casein kinase and cyclin-dependent kinase substrate, G two S phase expressed protein 1, prohibitin, nucleostemin, CDK2-associated protein 1 and block of proliferation 1 This is not a surprise since transformed cells grow more rapidly *in vitro *than during tumour development.

### Angiogenesis-involved genes

Some proangiogenic genes such as angiopoietin-like 4, selectin, endothelin 1, angiopoietin 2 and endothelial PAS domain protein 1 were up-regulated during tumour growth whereas the antiangiogenic factor thrombospondin 1 was found to be down regulated. Surprisingly, the proangiogenic endothelial cell growth factor 1 (platelet-derived) and the angiomotin like 2 were found down-regulated.

### Cytoskeleton and cell-to-cell contact genes

Expression of some genes involved in cytoskeleton and cell-to-cell contact was modified in tumours. mRNAs encoding junctophilin 3, fibrillin 2, plexin A3, ARP1 actin-related protein 1 homolog A, MYOSIN-IXA homolog, myosin IB, tubulin alpha 1, cadherin 3, integrin beta 5, dynamin and stathmin-like 4 genes were up-regulated whereas mRNAs encoding MAP/microtubule affinity-regulating kinase 2, nucleoporin 54 kDa, smooth muscle cell associated protein-1, plakophilin 2, annexin A3, myosin X, ARP1 actin-related protein 1 homolog B (yeast), follistatin, golgi associated gamma adaptin ear containing ARF binding protein 2, filamin beta, vinculin, cytoskeleton-associated protein 1, annexin A11, lamin A, cortactin, pericentrin 2, gap junction membrane channel protein alpha 1, importin 4, nucleolar protein 5, exportin 7, vinculin, actinin alpha 1, nucleoporin 88, fibulin 2 and CD44 antigen were down regulated.

### Extracellular matrix compounds

In cancer, transformed and mesenchymal cells synthesize and secrete several compounds which participate to tumour organization. In tumour cells, genes encoding the procollagen type XVIII alpha 1, Nice-4 protein homolog isoform 1, glycophorin A, collagen type V alpha 1 and procollagen type VI alpha 1 were up-regulated whereas, to our surprise, procollagen type V alpha 2 was found down regulated.

### Metabolic enzymes and secretory factors

Finally, an interesting finding is that the majority of genes encoding enzymes involved in metabolism were down regulated in tumours whereas the majority of secretory factors or their receptors were up-regulated (see additional files 1 and 2) suggesting some reduction of intracellular metabolic activity and increased signal exchanges during tumour formation.

## Conclusion

The microenvironment of the metastatic cancer cell and the interaction between these cells and the stroma play critical roles in tumour development and progression. However, the molecular mechanisms and genes involved in tumour development remain largely unidentified. In the experimental model of tumour formation used in this study, we identified 489 genes whose expression is modified during tumour formation. Among them, 213 were up-regulated and 276 were down-regulated. These genes are involved in a variety of cellular functions, including control of transcription, mRNA processing, regulation of translation, activation of some interferon-induced genes, intracellular signalling, apoptosis, cell growth, angiogenesis, cytoskeleton, cell adhesion, extracellular matrix formation, metabolism and production of secretory factors. These results can be interpreted in two ways *i/ *many cellular functions need to be adapted to allow successful tumour development or *ii/ *successful tumour formation has induced changes in gene expression. In fact, both interpretations are probably correct but the important point is that among them are found the genes involved in adaptation of the cancer cell to a new environment, which are potential targets for cancer therapy. This study therefore suggests that, after screening ~12,000 genes, the most interesting candidates for clinical applications are among the 213 genes up-regulated in the tumour.

## Material and Methods

### Transformation of MEFs by retroviral infection

Primary embryo fibroblasts were isolated from 14.5 day-old SV129J mouse embryos and grown in Dulbecco's modified Eagle's medium supplemented with 10% foetal calf serum as previously described [7]. We transduced MEFs with the pBabe-ras^V12^/E1A retroviral vector which expresses both the ras^V12 ^mutated protein and the E1A oncogene to obtain transformed fibroblasts. pBabe-ras^V12^/E1A plasmids were obtained from S. Lowe. Bosc 23 ecotropic packaging (10^6^) cells were plated in a 6-well plate, incubated for 24 hr, and then transfected with PEI with 5 μg of retroviral plasmid. After 48 hr, the medium containing the virus was filtered (0.45 μm filter, Millipore) to obtain the retroviral supernatant. MEFs were plated at 2 × 10^5 ^cells per 35 mm dish and incubated overnight. For infection, the culture medium was replaced by an appropriate mix of the retroviral supernatant and culture medium (V/V), supplemented with 4 μg/ml polybrene (Sigma), and cells were incubated at 37°C. Transformation of MEFs by the pBabe-ras^V12^/E1A retroviral vector was evaluated by examining changes in their morphological aspect, by quantifying expression of the RAS protein by western blot, by monitoring cell proliferation, colony formation in soft-agar and tumours in nude mice as previously described [7].

### Tumour induction in athymic mice

Suspensions of the pBabe-ras^V12^/E1A transformed MEFs (10^6^/200 μl PBS) were injected subcutaneously into the flank of male 8 week-old nu/nu mice, and tumours were allowed to develop for 20 days. Tumours were removed and stored at -80°C. Microscopical analysis reveals that tumours contain about 15% of vascular and stromal cells.

### Microarray

Total RNA from ras^V12^/E1A-transformed cells and tumours from three independent experiments was isolated by Trizol (Gibco-BRL). Twenty μg of total RNA was converted to cDNA with SuperScript reverse transcriptase (Gibco-BRL), using T7-oligo-d(T)_24 _as a primer. Second-strand synthesis was performed using T4 DNA polymerase and E. coli DNA ligase followed by blunt ending by T4 polynucleotide kinase. cDNA was isolated by phenol-chloroform extraction using phase lock gels (Brinkmann). cDNA was transcribed *in vitro *using the T7 BioArray High Yield RNA Transcript Labeling Kit (Enzo Biochem, New York, N.Y.) to produce biotinylated cRNA. Labelled cRNA was isolated using an RNeasy Mini Kit column (Qiagen). Purified cRNA was fragmented to 200–300 mer cRNA using a fragmentation buffer (100 mM potassium acetate-30 mM magnesium acetate-40 mM Tris-acetate, pH 8.1), for 35 min at 94°C. The quality of total RNA, cDNA synthesis, cRNA amplification and cRNA fragmentation was monitored by micro-capillary electrophoresis (Bioanalizer 2100, Agilent Technologies). The cRNA probes were hybridized to an MG u74Av2 Genechip (Affymetrix, Santa Clara, CA). Fifteen micrograms of fragmented cRNA was hybridized for 16 h at 45°C with constant rotation (60 rpm). Microarrays were processed in an Affymetrix GeneChip Fluidic Station 400. Staining was made with streptavidin-conjugated phycoerythrin (SAPE) followed by amplification with a biotinylated anti-streptavidin antibody and a second round of SAPE, and then scanned using an Agilent GeneArray Scanner (Agilent Technologies). The signal intensities for the β-actin and GAPDH genes were used as internal quality controls. The ratio of fluorescent intensities for the 5' and 3' ends of these housekeeping genes was <2. Scanned images were analyzed with the Microarray Suite 5.0 software (Affymetrix).

### Validation of gene expression profiles

One microgram of total RNA from primary embryo fibroblasts, ras^V12^/E1A transformed MEF and its derived tumour was subjected to PCR with reverse transcription using the One Step RT-PCR kit (Promega) according to the manufacturer's protocol. Selected RNA species were amplified using the following primers: arginase 1, sense, 5'-gaaaaggccgattcacctgag-3' and antisense, 5'-atgtggcgcattcacagtcac-3'; bone morphogenetic protein 10, sense, 5'-ggatctggacctggactcaga-3' and antisense, 5'-gaagctttctgggaattcttg-3'; cathepsin S, sense, 5'-gaagggctgcgtcactgaggt-3' and antisense, 5'-acaccgcttttgtagaagaag-3'; insulin-like growth factor binding protein 4, sense, 5'-gaaggtgtagagtagaggctc-3' and antisense, 5-ggaccagaatggggccattcc-3'; interferon stimulated gene 12, sense, 5'-ctcaacatgttgggaacactg-3' and antisense, 5-catctcctgcgtagtctgtac-3'; serum deprivation response, sense, 5'-gtctagtattataacctaacc-3' and antisense, 5-aagagtagagagttcgagccc-3'; thrombomodulin, sense, 5'-cagaaatttcaggtaaccaaa-3' and antisense, 5-tcagctcggcacgaagcacac-3'; adenylate kinase 1, sense, 5'-cactgggtgccaaggagctgt-3' and antisense, 5-ggcttcctgtgtaatgagacc-3'; connective tissue growth factor, sense, 5'-ggagtcagagccttgtctgtt-3' and antisense, 5-agtcataatcaaagaagcagc-3'; and S100 calcium binding protein A11, sense, 5'-gctgttttccaaaagtacagc-3' and antisense, 5-cgcttctgggaagtttggatg-3'. Reverse transcription was carried out 45 min at 48°C followed by 25–32 cycles of PCR, each cycle consisting in a denaturing step for 1 min at 94°C, an annealing step for 2 min at 56°C, and a polymerization step for 2 min at 72°C. PCR products were separated on a 1.0% agarose gel containing ethidium bromide and photographed under ultraviolet light.

## Authors' contributions

SV prepared cells and retroviruses, CM carried out RNA purification and RT-PCR analysis, ELC was in charge of microarray hybridization, JCD participated in the design of the study, JLI participated in the analysis of data and wrote the manuscript.

## Supplementary Material

Additional File 1 **Genes up-regulated during tumour development**. Genes found up-regulated by microarray analysis are listed, with their GenBank accession number, the over-expression factors (relative to ras^V12^/E1A transformed MEFs) observed in three separate experiments. Click here for file

Additional File 2 **Genes down-regulated during tumour development**. Genes found down-regulated by microarray analysis are listed, with their GenBank accession number, the down-regulation factors (relative to ras^V12^/E1A transformed MEFs) observed in three separate experiments. Click here for file
