# Supplementary material for: Gene expression profiling of tumours derived from rasV12/E1A-transformed mouse embryonic fibroblasts to identify genes required for tumour development
Source: Mol Cancer. 2005 Jan 16;4:4. doi: 10.1186/1476-4598-4-4 (PMC546195; doi:10.1186/1476-4598-4-4)
Supplement: Additional File 1 — Genes up-regulated during tumour development. Genes found up-regulated by microarray analysis are listed, with their GenBank accession number, the over-expression factors (relative to rasV12/E1A transformed MEFs) observed in three separate experiments. [file 1476-4598-4-4-S1.doc]

**Table 1 :** Up-regulated genes during cancer development

| **Descriptions** | **Fold up-regulated** | | **Accession number** |
| --- | --- | --- | --- |
| complement component 1, q subcomponent, beta polypeptide | 73.92 |  | M22531 |
| lectin, galactose binding, soluble 7 | 65.40 |  | AF038562 |
| hemoglobin, beta adult major chain | 52.58 |  | J00413 |
| hemoglobin, beta adult major chain | 48.98 |  | V00722 |
| apolipoprotein E | 48.52 |  | D00466 |
| complement component 1, q subcomponent, alpha polypeptide | 47.35 |  | X58861 |
| interferon-induced protein with tetratricopeptide repeats 1 | 44.56 |  | U43084 |
| arginase 1, liver | 40.27 |  | U51805 |
| TYRO protein tyrosine kinase binding protein | 38.63 |  | AF024637 |
| ubiquitin specific protease 18 | 33.44 |  | AW047653 |
| ADP-ribosylation factor-like 4 | 28.81 |  | D18912 |
| bone morphogenetic protein 10 | 28.53 |  | AF101440 |
| ARF-GAP, RHO-GAP, ankyrin repeat and pleckstrin homology domains-containing protein 3 | 25.97 |  | AI851258 |
| interferon-induced protein with tetratricopeptide repeats 3 | 24.28 |  | U43086 |
| guanosine diphosphate (GDP) dissociation inhibitor 2 | 23.78 |  | U07951 |
| complement component 1, q subcomponent, gamma polypeptide | 21.36 |  | X66295 |
| viral hemorrhagic septicemia virus(VHSV) induced gene 1 | 20.64 |  | AA204579 |
| SET domain, bifurcated 1 | 19.20 |  | C77070 |
| acid phosphatase 5, tartrate resistant | 18.57 |  | M99054 |
| serine (or cysteine) proteinase inhibitor, clade B, member 9 | 18.32 |  | U96700 |
| cathepsin S | 15.87 |  | AJ223208 |
| insulin-like growth factor binding protein 4 | 15.58 |  | AI838737 |
| interferon regulatory factor 7 | 14.94 |  | U73037 |
| hemoglobin alpha, adult chain 1 | 14.60 |  | V00714 |
| guanylate nucleotide binding protein 3 | 14.31 |  | AW047476 |
| EST | 12.47 |  | C78246 |
| CCR4-NOT transcription complex, subunit 7 | 12.30 |  | AI931748 |
| EST | 12.17 |  | C78041 |
| EST | 11.28 |  | C79086 |
| B-cell CLL/lymphoma 6, member B | 10.85 |  | AB011665 |
| interferon stimulated gene 12 | 10.45 |  | AI158810 |
| SH3-domain GRB2-like 2 | 10.36 |  | U58886 |
| EST | 9.89 |  | M32486 |
| olfactory receptor 136 | 9.70 |  | AJ132195 |
| ephrin A2 | 9.22 |  | U14941 |
| glutamate receptor, ionotropic, delta 2 | 8.74 |  | D13266 |
| apolipoprotein A-II | 8.71 |  | X62772 |
| proteosome (prosome, macropain) subunit, beta type 8 (large multifunctional protease 7) | 8.69 |  | U22033 |
| EST | 8.32 |  | C78037 |
| interferon, alpha-inducible protein | 7.18 |  | AV152244 |
| tissue factor pathway inhibitor | 6.97 |  | AF004833 |
| elongation of very long chain fatty acids (FEN1/Elo2, SUR4/Elo3, yeast)-like 2 | 6.70 |  | AI317360 |
| EST | 6.46 |  | AV096879 |
| EST | 6.45 |  | AV292380 |
| EST | 6.43 |  | AI448172 |
| interferon consensus sequence binding protein 1 | 6.34 |  | M32489 |
| EST | 6.26 |  | AV374010 |
| EST | 6.22 |  | AV064697 |
| EST | 6.12 |  | AA607237 |
| EST | 6.10 |  | AV216468 |
| melanoma cell adhesion molecule | 5.94 |  | AI853261 |
| EST | 5.90 |  | AA438122 |
| CD52 antigen | 5.70 |  | M55561 |
| hemoglobin alpha, adult chain 1 | 5.61 |  | AV003378 |
| interferon-inducible GTPase | 5.47 |  | AA914345 |
| rap2 interacting protein x | 5.47 |  | AW049573 |
| REST corepressor 1 | 5.40 |  | AV356223 |
| chemokine (C-X-C motif) receptor 4 | 5.36 |  | Z80112 |
| EST | 5.36 |  | C78228 |
| junctophilin 3 | 5.29 |  | AI836349 |
| EST | 5.26 |  | AI851206 |
| EST | 5.24 |  | AV153195 |
| interferon induced transmembrane protein 2 | 4.93 |  | AI790103 |
| Williams-Beuren syndrome chromosome region 5 homolog | 4.92 |  | AW125574 |
| glial cells missing homolog 2 (Drosophila) | 4.92 |  | D88611 |
| stanniocalcin 1 | 4.89 |  | U47815 |
| inhibitor of DNA binding 2 | 4.86 |  | AF077861 |
| transcription factor 15 | 4.82 |  | U18658 |
| lymphocyte cytosolic protein 2 | 4.78 |  | U20159 |
| TGFB inducible early growth response 1 | 4.75 |  | AF064088 |
| spermatogenesis apoptosis-related protein | 4.74 |  | AV111954 |
| EST | 4.65 |  | AV315398 |
| fibrillin 2 | 4.63 |  | L39790 |
| apolipoprotein B editing complex 1 | 4.55 |  | U22262 |
| Rhesus blood group-associated A glycoprotein | 4.52 |  | AF057527 |
| calpain 5 | 4.46 |  | Y10656 |
| dimerization cofactor of hepatocyte nuclear factor 1 alpha (TCF1) | 4.36 |  | AW046590 |
| pleckstrin homology domain containing, family A (phosphoinositide binding specific) member 1 | 4.35 |  | AV335799 |
| mesoderm specific transcript | 4.34 |  | AF017994 |
| EST | 4.31 |  | C79108 |
| A kinase (PRKA) anchor protein 8 | 4.25 |  | AV292740 |
| EST | 4.24 |  | AI158971 |
| EST | 4.21 |  | AA407332 |
| PDZ and LIM domain 4 | 4.13 |  | Y08361 |
| BCL2/adenovirus E1B 19kDa-interacting protein 1, NIP3 | 4.13 |  | AF041054 |
| protein tyrosine phosphatase, non-receptor type 13 | 4.12 |  | D83966 |
| myxovirus (influenza virus) resistance 2 | 4.11 |  | J03368 |
| angiopoietin-like 4 | 4.07 |  | AA797604 |
| EST | 4.05 |  | AW046850 |
| schlafen 4 | 4.04 |  | AF099974 |
| early growth response 2 | 4.04 |  | M24377 |
| chromobox homolog 3 (Drosophila HP1 gamma) | 3.98 |  | X56683 |
| EST | 3.90 |  | C77335 |
| EST | 3.89 |  | AV296708 |
| EST | 3.87 |  | AA794923 |
| calcium and integrin binding family member 2 | 3.86 |  | AB016080 |
| EST | 3.82 |  | AV282092 |
| interferon gamma induced GTPase | 3.81 |  | U53219 |
| diaphanous homolog 2 (Drosophila) | 3.80 |  | Y15910 |
| factor in the germline alpha | 3.80 |  | U91840 |
| selectin, endothelial cell | 3.79 |  | M80778 |
| EST | 3.78 |  | AW046694 |
| vacuolar protein sorting 35 | 3.78 |  | AV266447 |
| Xlr-related, meiosis regulated | 3.78 |  | X72697 |
| EST | 3.73 |  | AV055186 |
| ets variant gene 1 | 3.72 |  | L10426 |
| CXXC finger 5 | 3.71 |  | AW124069 |
| Igkappa gene for immunoglobulin kappa chain, partial cds, strain:BDF1. | 3.69 |  | U30241 |
| SH3-domain GRB2-like 3 | 3.67 |  | U58887 |
| EST | 3.62 |  | AV360058 |
| immunoglobulin heavy chain (V10 family) | 3.61 |  | Z70661 |
| lectin, mannose-binding 2 | 3.60 |  | AV019864 |
| TGFB inducible early growth response 1 | 3.52 |  | AF064088 |
| chemokine-like factor super family 8 | 3.47 |  | AI787137 |
| EST | 3.46 |  | AI642417 |
| EST | 3.45 |  | AI845581 |
| tryptophan hydroxylase 1 | 3.45 |  | AV250738 |
| Diabetic nephropathy-related gene 1 mRNA, partial sequence | 3.45 |  | AA816121 |
| interferon-g induced GTPase | 3.43 |  | AJ007972 |
| basic helix-loop-helix domain containing, class B2 | 3.41 |  | Y07836 |
| pre-B-cell colony-enhancing factor 1 | 3.39 |  | Y17860 |
| retinoblastoma-associated factor 600 | 3.38 |  | AV086272 |
| endothelin 1 | 3.37 |  | U35233 |
| interleukin 1 receptor-like 1 | 3.32 |  | D13695 |
| EST | 3.24 |  | AI851014 |
| Janus kinase 3 | 3.24 |  | AV266650 |
| interferon, gamma-inducible protein 16 | 3.20 |  | M31419 |
| EST | 3.10 |  | AV349142 |
| EST | 3.08 |  | C81070 |
| human immunodeficiency virus type I enhancer binding protein 2 | 3.02 |  | Y15907 |
| plexin A3 | 2.94 |  | D86950 |
| dual specificity phosphatase 9 | 2.93 |  | AV225351 |
| EST | 2.92 |  | C79764 |
| procollagen, type XVIII, alpha 1 | 2.90 |  | D17546 |
| receptor tyrosine kinase-like orphan receptor 2 | 2.89 |  | AI596034 |
| cathepsin K | 2.87 |  | AJ006033 |
| heterogeneous nuclear ribonucleoprotein U | 2.83 |  | AA981581 |
| Nik related kinase | 2.82 |  | AA866768 |
| Nice-4 protein homolog isoform 1 | 2.82 |  | C77404 |
| homeo box C6 | 2.81 |  | M35986 |
| tumor necrosis factor receptor superfamily, member 11b (osteoprotegerin) | 2.80 |  | U94331 |
| synaptojanin 2 | 2.79 |  | AF041859 |
| EST | 2.79 |  | AI837905 |
| cyclin-dependent kinase 7 (homolog of Xenopus MO15 cdk-activating kinase) | 2.77 |  | X74145 |
| serine (or cysteine) proteinase inhibitor, clade A, member 1a | 2.69 |  | M25529 |
| zinc finger, RAN-binding domain containing 1 | 2.68 |  | AA981257 |
| solute carrier family 30 (zinc transporter), member 1 | 2.68 |  | U17132 |
| complement component 2 (within H-2S) | 2.68 |  | AV233144 |
| antigen identified by monoclonal antibody MRC OX-2 | 2.66 |  | AF029215 |
| interleukin 13 receptor, alpha 1 | 2.65 |  | AA608387 |
| ARP1 actin-related protein 1 homolog A | 2.65 |  | AB010297 |
| angiopoietin 2 | 2.65 |  | AF004326 |
| netrin 1 | 2.64 |  | AA645293 |
| calpain 6 | 2.63 |  | AI747133 |
| crystallin, gamma S | 2.63 |  | AF032995 |
| EST | 2.62 |  | AA794350 |
| EST | 2.61 |  | C77776 |
| complement component 1, r subcomponent | 2.61 |  | AI132585 |
| glycophorin A | 2.61 |  | M26385 |
| MYOSIN-IXA homolog | 2.59 |  | AW125669 |
| collagen, type V, alpha 1 | 2.56 |  | AA796989 |
| inhibitor of growth family, member 4 | 2.56 |  | AI845183 |
| ERO1-like (S. cerevisiae) | 2.54 |  | AA798624 |
| inositol hexaphosphate kinase 1 | 2.54 |  | AW123807 |
| proteasome (prosome, macropain) subunit, beta type 10 | 2.54 |  | Y10875 |
| BTB (POZ) domain containing 1 | 2.52 |  | AI848446 |
| Ig kappa chain V-V region L7 precursor (Fragment) | 2.51 |  | X88903 |
| T-cell receptor alpha chain | 2.51 |  | AF099808 |
| galactose mutarotase | 2.50 |  | AV367320 |
| amylase 2, pancreatic | 2.50 |  | X02578 |
| killer cell lectin-like receptor subfamily C, member 1 | 2.49 |  | AF095447 |
| wingless related MMTV integration site 10a | 2.49 |  | U61969 |
| NAD(P) dependent steroid dehydrogenase-like | 2.48 |  | AW106745 |
| dimethylarginine dimethylaminohydrolase 2 | 2.47 |  | AF004106 |
| spleen tyrosine kinase | 2.47 |  | U25685 |
| EST | 2.46 |  | C80249 |
| erythrocyte protein band 7.2 | 2.46 |  | U17297 |
| neoplastic progression 1 | 2.45 |  | Z31360 |
| procollagen, type VI, alpha 1 | 2.44 |  | X66405 |
| ribitol kinase, putative | 2.44 |  | AI131744 |
| myosin IB | 2.43 |  | L00923 |
| cytochrome c oxidase, subunit VIIc | 2.43 |  | AI648091 |
| B lymphoid kinase | 2.42 |  | AA204265 |
| tubulin, alpha 1 | 2.40 |  | M28729 |
| hnRNP-associated with lethal yellow | 2.40 |  | L17076 |
| DEAD (Asp-Glu-Ala-Asp) box polypeptide 27 | 2.40 |  | AV250694 |
| TAP binding protein | 2.39 |  | AF110520 |
| mitogen activated protein kinase kinase kinase kinase 1 | 2.38 |  | Y09010 |
| EST | 2.38 |  | AI850438 |
| endothelial PAS domain protein 1 | 2.37 |  | AF045160 |
| cholecystokinin B receptor | 2.35 |  | AF019371 |
| Janus kinase 3 | 2.35 |  | L40172 |
| cadherin 3 | 2.33 |  | AV322859 |
| MADP-1 protein | 2.32 |  | AI845977 |
| integrin beta 5 | 2.31 |  | AV106844 |
| EST | 2.31 |  | AV245978 |
| histocompatibility 2, T region locus 18 | 2.29 |  | X03052 |
| EST | 2.26 |  | AI842970 |
| dynamin | 2.26 |  | L31397 |
| EST | 2.26 |  | AA914734 |
| immunoglobulin (CD79A) binding protein 1 | 2.25 |  | AV048486 |
| zinc finger protein 37 | 2.24 |  | X52533 |
| membrane-spanning 4-domains, subfamily A, member 1 | 2.23 |  | AA797989 |
| stathmin-like 4 | 2.22 |  | AF105222 |
| enhancer of zeste homolog 1 (Drosophila) | 2.19 |  | U60453 |
| solute carrier family 16 (monocarboxylic acid transporters), member 2 | 2.18 |  | AF045692 |
| EST | 2.17 |  | AI117835 |
| peptidylprolyl isomerase (cyclophilin)-like 2 | 2.17 |  | AW122632 |
| solute carrier family 34 (sodium phosphate), member 1 | 2.17 |  | L33878 |
| CXORF15 | 2.16 |  | AA796133 |
| tumor-associated calcium signal transducer 1 | 2.14 |  | M76124 |
| AT rich interactive domain 3B (Bright like) | 2.08 |  | AF1168479 |
| ral guanine nucleotide dissociation stimulator | 2.06 |  | L07924 |
