# Supplementary material for: Gene expression profiling of tumours derived from rasV12/E1A-transformed mouse embryonic fibroblasts to identify genes required for tumour development
Source: Mol Cancer. 2005 Jan 16;4:4. doi: 10.1186/1476-4598-4-4 (PMC546195; doi:10.1186/1476-4598-4-4)
Supplement: Additional File 2 — Genes down-regulated during tumour development. Genes found down-regulated by microarray analysis are listed, with their GenBank accession number, the down-regulation factors (relative to rasV12/E1A transformed MEFs) observed in three separate experiments. [file 1476-4598-4-4-S2.doc]

**Table 2 :** Down-regulated genes during cancer development

| **Descriptions** | **Folds down regulated** | **Accession number** | |
| --- | --- | --- | --- |
| connective tissue growth factor | 80.66 |  | M70642 |
| adenylate kinase 1 | 40.00 |  | AJ010108 |
| ephrin B2 | 20.02 |  | U30244 |
| zinc finger protein 36, C3H type-like 2 | 19.13 |  | M58564 |
| DEAD/DEAH box helicase, putative | 18.21 |  | AV338120 |
| serum deprivation response | 17.27 |  | AI839175 |
| MAP/microtubule affinity-regulating kinase 2 | 15.97 |  | X70764 |
| excision repair cross-complementing rodent repair deficiency,complementation group 5 | 15.40 |  | U40796 |
| phosphomannomutase 1 | 14.76 |  | AF007267 |
| forkhead box M1 | 13.43 |  | Y11245 |
| growth arrest and DNA-damage-inducible 45 beta | 12.75 |  | AV138783 |
| early B-cell factor 3; Olf-1/EBF-like 2 [Mus musculus] | 11.87 |  | AI043090 |
| HLA-B-associated transcript 3 | 11.39 |  | AW047616 |
| ATP-binding cassette, sub-family F (GCN20), member 2 | 11.35 |  | AI837318 |
| glucose-6-phosphate dehydrogenase X-linked | 11.34 |  | Z84471 |
| cysteine rich protein 61 | 11.17 |  | M32490 |
| solute carrier family 6 (neurotransmitter transporter, taurine), member 6 | 10.90 |  | AI042802 |
| protein kinase C, alpha | 10.80 |  | M25811 |
| dehydrogenase/reductase (SDR family) member 4 | 10.21 |  | AW260761 |
| nucleoporin 54kDa | 9.93 |  | AA683712 |
| neural proliferation, differentiation and control gene 1 | 9.61 |  | X67209 |
| Zic family member 1 (odd-paired homolog, Drosophila) | 9.60 |  | D32167 |
| RIO kinase 1 (yeast) | 9.56 |  | AI172819 |
| acyl-Coenzyme A thioesterase 2, mitochondrial | 9.52 |  | AI854821 |
| eukaryotic translation initiation factor 4, gamma 1 | 9.28 |  | AV380793 |
| T-box 14 | 9.14 |  | AF013282 |
| protein phosphatase 1, regulatory (inhibitor) subunit 2 | 9.13 |  | AW049584 |
| phosphotidylinositol transfer protein, beta | 8.75 |  | AI747899 |
| nerve growth factor, beta | 8.60 |  | M17298 |
| RAN GTPase activating protein 1 | 8.28 |  | U20857 |
| dihydrolipoamide branched chain transacylase E2 | 8.26 |  | L42996 |
| PR domain containing 5 | 8.16 |  | AI314715 |
| meiotic recombination 11 homolog A (S. cerevisiae) | 8.06 |  | U60318 |
| complement receptor related protein | 7.77 |  | M23529 |
| EST | 7.73 |  | AW123514 |
| Eph receptor A2 | 7.67 |  | U07634 |
| SET and MYND domain containing 2 | 7.61 |  | AI847158 |
| ER degradation enhancer, mannosidase alpha-like 1 | 7.50 |  | AW212878 |
| thrombomodulin | 7.45 |  | AV364086 |
| DC12 protein | 7.43 |  | AA930519 |
| paraspeckle protein 1 | 7.43 |  | AA738776 |
| CUG triplet repeat, RNA binding protein 1 | 7.42 |  | X61451 |
| copper chaperone for superoxide dismutase | 7.34 |  | AI839702 |
| v-crk sarcoma virus CT10 oncogene homolog (avian)-like | 7.17 |  | X90648 |
| X-ray repair complementing defective repair in Chinese hamster cells 5 | 7.10 |  | X66323 |
| serum/glucocorticoid regulated kinase | 7.06 |  | AW046181 |
| EST | 7.04 |  | AW124941 |
| EST | 7.02 |  | AW125433 |
| uridine monophosphate synthetase | 6.89 |  | M29395 |
| smooth muscle cell associated protein-1 | 6.84 |  | AW050153 |
| DEAD (Asp-Glu-Ala-Asp) box polypeptide 48 | 6.80 |  | AW124859 |
| plakophilin 2 | 6.79 |  | AW208938 |
| splicing factor, arginine/serine-rich 2, interacting protein | 6.77 |  | AW123947 |
| neoplastic progression 3 | 6.77 |  | Z31362 |
| lactamase, beta 2 | 6.75 |  | AI841387 |
| ilvB (bacterial acetolactate synthase)-like | 6.65 |  | AI842668 |
| annexin A3 | 6.51 |  | AJ001633 |
| nucleolar GTPase | 6.50 |  | AI853259 |
| HDCMA18P protein | 6.49 |  | AW122781 |
| ribosomal protein S6 kinase, polypeptide 4 | 6.38 |  | AF074714 |
| calcium/calmodulin-dependent protein kinase II, delta | 6.33 |  | AF059029 |
| EST | 6.31 |  | AW107973 |
| sine oculis-related homeobox 1 homolog (Drosophila) | 6.29 |  | X80339 |
| outer dense fiber of sperm tails 2 | 6.29 |  | AF034105 |
| sorting nexin 14 | 6.24 |  | AI839611 |
| aminopeptidase-like 1 | 6.12 |  | AA726383 |
| liver-specific bHLH-Zip transcription factor | 6.06 |  | U49507 |
| propionyl Coenzyme A carboxylase, beta polypeptide | 6.02 |  | AA882332 |
| EST | 5.89 |  | AI847050 |
| deformed epidermal autoregulatory factor 1 (Drosophila) | 5.76 |  | AF102818 |
| HMG (high mobility group) box, Bromodomain (5 domains), Zinc finger, C2H2 type | 5.64 |  | AW048644 |
| myosin X | 5.63 |  | AJ249706 |
| cyclin G1 | 5.59 |  | L49507 |
| EST | 5.56 |  | AV214912 |
| sorting nexin 17 | 5.53 |  | AW123761 |
| EST | 5.50 |  | AW209561 |
| EST | 5.46 |  | AI642561 |
| cellular retinoic acid binding protein I | 5.37 |  | X15789 |
| AFG3(ATPase family gene 3)-like 1 (yeast) | 5.23 |  | AA797556 |
| wild-type p53-induced gene 1 | 5.19 |  | AF012923 |
| transforming growth factor beta regulated gene 4 | 5.17 |  | U89434 |
| bone morphogenetic protein receptor, type 1A | 5.13 |  | D16250 |
| EST | 5.11 |  | AI836641 |
| EST | 5.11 |  | AA657044 |
| EST | 5.11 |  | AI850923 |
| enabled homolog (Drosophila) | 5.09 |  | U72521 |
| amyloid beta (A4) precursor protein-binding, family B, member 2 | 5.08 |  | U70210 |
| ARP1 actin-related protein 1 homolog B (yeast) | 5.01 |  | AI843424 |
| thrombospondin 1 | 5.00 |  | M62470 |
| Rho GDP dissociation inhibitor (GDI) gamma | 5.00 |  | U73198 |
| cytokine receptor-like factor 1 | 4.94 |  | AA270365 |
| S-adenosylmethionine decarboxylase 1 | 4.93 |  | Z23077 |
| spastic paraplegia 20, spartin (Troyer syndrome) homolog (human) | 4.75 |  | AA797709 |
| follistatin | 4.70 |  | Z29532 |
| cell division cycle 34 homolog (S. cerevisiae) | 4.65 |  | AW120683 |
| elongation protein 3 homolog (S. cerevisiae) | 4.59 |  | AI851229 |
| S100 calcium binding protein A11 (calizzarin) | 4.57 |  | U41341 |
| MEP50 protein | 4.55 |  | AI853476 |
| glycolipid transfer protein | 4.55 |  | AI842825 |
| protein-L-isoaspartate (D-aspartate) O-methyltransferase 1 | 4.53 |  | AW124044 |
| expressed in non-metastatic cells 4, protein | 4.51 |  | AI836205 |
| cornichon homolog (Drosophila) | 4.43 |  | AF022811 |
| butyrate-induced transcript 1 | 4.42 |  | Z97207 |
| golgi associated, gamma adaptin ear containing, ARF binding protein 2 | 4.37 |  | AW121839 |
| phosphatidylinositol 3-kinase, regulatory subunit, polypeptide 1 (p85 alpha) | 4.35 |  | U50413 |
| mitogen activated protein kinase 1 | 4.28 |  | D87271 |
| BTB (POZ) domain containing 14A | 4.27 |  | AW122114 |
| spermidine synthase | 4.22 |  | Z67748 |
| Jun oncogene | 4.22 |  | X12761 |
| EST | 4.18 |  | AA855382 |
| general transcription factor III A | 4.16 |  | AW210248 |
| IQ motif containing GTPase activating protein 1 | 4.13 |  | AI642553 |
| esterase 10 | 4.13 |  | AB025408 |
| EST | 4.12 |  | AA688938 |
| cyclin-dependent kinase inhibitor 1A (P21) | 4.12 |  | U09507 |
| Bcl2-associated X protein | 4.08 |  | L22472 |
| peroxiredoxin 6 | 3.97 |  | AF093853 |
| solute carrier family 19 (sodium/hydrogen exchanger), member 1 | 3.93 |  | L23755 |
| endothelial cell growth factor 1 (platelet-derived) | 3.83 |  | AW123987 |
| filamin, beta | 3.80 |  | AI838592 |
| vinculin | 3.78 |  | L18880 |
| eukaryotic translation initiation factor 5B | 3.78 |  | AA647048 |
| peptidylprolyl isomerase F (cyclophilin F) | 3.77 |  | AI842675 |
| protein kinase C, epsilon | 3.76 |  | AW120767 |
| cytoskeleton-associated protein 1 | 3.73 |  | AI853425 |
| EH-domain containing 1 | 3.72 |  | AI844128 |
| angiomotin like 2 | 3.68 |  | AI854404 |
| MYB binding protein (P160) 1a | 3.52 |  | AI506202 |
| small fragment nuclease | 3.50 |  | AI839882 |
| programmed cell death 2 | 3.47 |  | U10903 |
| annexin A11 | 3.45 |  | U65986 |
| lamin A | 3.41 |  | D49733 |
| cortactin | 3.38 |  | U03184 |
| ubiquitin-conjugating enzyme E2S | 3.37 |  | AI837415 |
| SGT1, suppressor of G2 allele of SKP1 (S. cerevisiae) | 3.33 |  | AI838149 |
| general transcription factor II H, polypeptide 1 | 3.31 |  | AJ002366 |
| transducin (beta)-like 3 | 3.30 |  | AA153773 |
| transcription factor Dp 1 | 3.28 |  | X72310 |
| PERP, TP53 apoptosis effector | 3.28 |  | AI854029 |
| pericentrin 2 | 3.20 |  | AI194767 |
| OGT(O-Glc-NAc transferase)-interacting protein 106 KDa | 3.20 |  | AI835632 |
| translocated promoter region | 3.18 |  | AW121876 |
| DnaJ (Hsp40) homolog, subfamily C, member 3 | 3.11 |  | U28423 |
| HLA-B associated transcript 2 | 3.09 |  | AW050268 |
| EST | 2.99 |  | AI049391 |
| serine/threonine kinase 16 | 2.97 |  | AF062076 |
| SEC14-like 1 (S. cerevisiae) | 2.96 |  | AI852087 |
| gap junction membrane channel protein alpha 1 | 2.96 |  | M63801 |
| cathepsin L | 2.95 |  | X06086 |
| 3-oxoacid CoA transferase 1 | 2.94 |  | AI843232 |
| DiGeorge syndrome critical region gene 6 | 2.93 |  | AF021031 |
| MIC2 (monoclonal Imperial Cancer Research Fund 2)-like 1 | 2.93 |  | AW050035 |
| cytidine monophospho-N-acetylneuraminic acid synthetase | 2.92 |  | AJ006215 |
| glutamate-cysteine ligase , modifier subunit | 2.91 |  | U95053 |
| Yeast SNF7 like protein | 2.91 |  | AW122839 |
| transcription factor Dp 1 | 2.89 |  | X72310 |
| myeloblastosis oncogene-like 2 | 2.88 |  | X70472 |
| lectin, galactose binding, soluble 3 | 2.87 |  | X16834 |
| nuclear, casein kinase and cyclin-dependent kinase substrate | 2.86 |  | AW048468 |
| G two S phase expressed protein 1 | 2.84 |  | AJ222580 |
| mitochondrial ribosomal protein L18 | 2.83 |  | AI837302 |
| mitochondrial isoleucine tRNA synthetase | 2.80 |  | AW048882 |
| thymoma viral proto-oncogene 1 | 2.79 |  | X65687 |
| purine-nucleoside phosphorylase | 2.76 |  | U35374 |
| brix domain-containing protein | 2.74 |  | AW124599 |
| chromosome condensation 1 | 2.73 |  | AA756292 |
| importin 4 | 2.71 |  | AI467390 |
| nucleolar protein 5 | 2.69 |  | AF053232 |
| upregulated during skeletal muscle growth 5 | 2.69 |  | AW060158 |
| general transcription factor IIF, polypeptide 1, 74kDa | 2.68 |  | AI853340 |
| superoxide dismutase 1, soluble | 2.67 |  | M35725 |
| exportin 7 | 2.65 |  | AI606243 |
| EST | 2.64 |  | AI853651 |
| programmed cell death 6 interacting protein | 2.64 |  | AI840810 |
| apoptotic chromatin condensation inducer 1 | 2.61 |  | AI839299 |
| 4-nitrophenylphosphatase domain and non-neuronal SNAP25-like protein homolog 1 (C. elegans) | 2.61 |  | AJ001260 |
| chemokine (C-X-C motif) ligand 1 | 2.61 |  | J04596 |
| ATP-binding cassette, sub-family B (MDR/TAP), member 7 | 2.61 |  | U43892 |
| nardilysin, N-arginine dibasic convertase, NRD convertase 1 | 2.60 |  | AA734806 |
| proteasome (prosome, macropain) 26S subunit, non-ATPase, 1 | 2.59 |  | AW123318 |
| COP9 (constitutive photomorphogenic) homolog, subunit 5 (Arabidopsis thaliana) | 2.59 |  | U70736 |
| small nuclear ribonucleoprotein polypeptide A | 2.59 |  | AW227345 |
| protein phosphatase 1, regulatory (inhibitor) subunit 7 | 2.59 |  | AI849126 |
| v-ets erythroblastosis virus E26 oncogene homolog 1 (avian) | 2.58 |  | AI882555 |
| prohibitin | 2.58 |  | X78682 |
| heat shock protein 105 | 2.57 |  | L40406 |
| polo-like kinase 2 (Drosophila) | 2.56 |  | M96163 |
| diaphanous homolog 3 (Drosophila) | 2.55 |  | AF094519 |
| vinculin | 2.54 |  | AI462105 |
| heat shock protein 1 (chaperonin 10) | 2.53 |  | U09659 |
| uridine monophosphate kinase | 2.53 |  | AI850362 |
| similar to ribosome-binding protein p34 | 2.52 |  | AW125178 |
| nucleostemin | 2.52 |  | AI785289 |
| procollagen, type V, alpha 2 | 2.52 |  | L02918 |
| tumor-associated antigen 1 | 2.52 |  | U35836 |
| translocase of inner mitochondrial membrane 23 homolog (yeast) | 2.51 |  | AB021122 |
| actinin, alpha 1 | 2.51 |  | AI195392 |
| nudix (nucleoside diphosphate linked moiety X)-type motif 14 | 2.50 |  | AW048282 |
| EST | 2.50 |  | AA675468 |
| dual specificity phosphatase 1 | 2.49 |  | X61940 |
| Rho-associated coiled-coil forming kinase 2 | 2.49 |  | U58513 |
| splicing factor 3b, subunit 1 | 2.48 |  | AI844532 |
| acid phosphatase 1, soluble | 2.48 |  | Y17343 |
| adenylate kinase 3 alpha-like | 2.47 |  | AB020203 |
| cell division cycle and apoptosis regulator 1 | 2.47 |  | AI035334 |
| transcriptional regulator, SIN3B (yeast) | 2.47 |  | AF038848 |
| EST | 2.46 |  | AI840541 |
| RNA polymerase I transcription factor RRN3 | 2.46 |  | AI838293 |
| Kruppel-like factor 5 | 2.46 |  | AA611766 |
| EST | 2.45 |  | AW125109 |
| synaptotagmin binding, cytoplasmic RNA interacting protein | 2.45 |  | AF093821 |
| Friend leukemia integration 1 | 2.44 |  | X59421 |
| proteasome (prosome, macropain) 26S subunit, non-ATPase, 7 | 2.44 |  | M64641 |
| mitsugumin 23 [Mus musculus] | 2.43 |  | AI853127 |
| nucleoporin 88 | 2.42 |  | AW120775 |
| mitochondrial processing peptidase beta | 2.41 |  | AI157548 |
| PAK1 interacting protein 1 | 2.41 |  | AI846025 |
| RAN binding protein 1 | 2.41 |  | X56045 |
| reticulocalbin 2 | 2.41 |  | AF049125 |
| S-adenosylmethionine decarboxylase 1 | 2.41 |  | D12780 |
| ADP-ribosylation factor-like 10C | 2.40 |  | AI286904 |
| general transcription factor III C 1 | 2.40 |  | AI836082 |
| fibulin 2 | 2.39 |  | X75285 |
| inosine 5'-phosphate dehydrogenase 1 | 2.37 |  | U00978 |
| translocating chain-associating membrane protein 1 | 2.37 |  | AA763937 |
| mitochondrial ribosomal protein L44 | 2.36 |  | AW124918 |
| EST | 2.36 |  | AI852970 |
| UDP-Gal:betaGlcNAc beta 1,3-galactosyltransferase, polypeptide 3 | 2.35 |  | AF029792 |
| arginine-rich, mutated in early stage tumors | 2.34 |  | AW122364 |
| tyrosine 3-monooxygenase/tryptophan 5-monooxygenase activation protein, zeta polypeptide | 2.33 |  | D83037 |
| coagulation factor II (thrombin) receptor | 2.33 |  | AW046032 |
| EST | 2.33 |  | AA177382 |
| nucleosome assembly protein 1-like 1 | 2.33 |  | X61449 |
| mitochondrial ribosomal protein S25 | 2.33 |  | C77227 |
| dual specificity phosphatase 16 | 2.33 |  | AI642662 |
| EST | 2.33 |  | AI847584 |
| molybdenum cofactor sulfurase family protein | 2.32 |  | AW124268 |
| RAB23, member RAS oncogene family | 2.32 |  | Z22821 |
| acid phosphatase 1, soluble | 2.31 |  | Y17345 |
| Hypothetical NOL1/NOP2/sun family containing protein | 2.31 |  | AI787627 |
| mitochondrial ribosomal protein S10 | 2.31 |  | AI842124 |
| ornithine decarboxylase antizyme inhibitor | 2.31 |  | AF032128 |
| WD repeat domain 26 | 2.30 |  | AA795284 |
| eukaryotic translation initiation factor 3, subunit 1 alpha | 2.30 |  | AI848056 |
| CDK2 (cyclin-dependent kinase 2)-associated protein 1 | 2.29 |  | AF011644 |
| mannose-6-phosphate receptor, pseudogene | 2.29 |  | X56831 |
| PDZ and LIM domain 1 (elfin) | 2.29 |  | AF053367 |
| EST | 2.28 |  | AW212071 |
| proviral integration site 3 | 2.28 |  | AW046627 |
| calcium channel, voltage-dependent, alpha2/delta subunit 1 | 2.26 |  | U73487 |
| eukaryotic translation initiation factor 1A | 2.26 |  | AF026481 |
| EST | 2.26 |  | AW125724 |
| general transcription factor IIB | 2.24 |  | AI840450 |
| transformation related protein 53 (p53) | 2.24 |  | AB021961 |
| procollagen-lysine, 2-oxoglutarate 5-dioxygenase 3 | 2.23 |  | AW107836 |
| block of proliferation 1 | 2.21 |  | AF061503 |
| glycine C-acetyltransferase (2-amino-3-ketobutyrate-coenzyme A ligase) | 2.21 |  | AI849354 |
| NADH dehydrogenase (ubiquinone) Fe-S protein 2 | 2.20 |  | AI837493 |
| EST | 2.19 |  | AI648018 |
| CD44 antigen | 2.18 |  | X66084 |
| ornithine decarboxylase, structural | 2.18 |  | M12330 |
| mitogen activated protein kinase kinase 4 | 2.16 |  | U18310 |
| COP9 (constitutive photomorphogenic) homolog, subunit 2 (Arabidopsis thaliana) | 2.14 |  | AF071312 |
| RNA-binding region (RNP1, RRM) containing 1 | 2.14 |  | X75316 |
| adenosine kinase | 2.13 |  | AW121801 |
| large tumor suppressor 2 | 2.12 |  | AI849416 |
| caspase 7 | 2.10 |  | U67321 |
| caspase 7 | 2.10 |  | D86353 |
| myeloid-associated differentiation marker | 2.10 |  | AJ001616 |
| acetyl-Coenzyme A dehydrogenase, long-chain | 2.09 |  | U21489 |
| DEAD (Asp-Glu-Ala-Asp) box polypeptide 21 | 2.07 |  | AI836083 |
| DNAJ domain-containing | 2.07 |  | AA754865 |
| nucleosome assembly protein 1-like 1 | 2.07 |  | AW107230 |
| lysophospholipase 1 | 2.07 |  | AA840463 |
| Der1-like family protein / degradation in the ER-like family protein | 2.05 |  | AW120606 |
| NADH dehydrogenase (ubiquinone) flavoprotein 2 | 2.01 |  | AI847609 |
